# Fellowships, Grants, & Awards

**Published:** 2007-09

## Advancing Novel Science in Women’s Health Research (ANSWHR) [R21]

The Office of Research on Women’s Health (ORWH), established in 1990 within the Office of the Director, National Institutes of Health (NIH), *a*) advises the NIH Director and staff on matters relating to research on women’s health; *b*) strengthens and enhances research related to diseases, disorders, and conditions that affect women; *c*) ensures that research conducted and supported by NIH adequately addresses issues regarding women’s health; *d*) ensures that women are appropriately represented in biomedical and biobehavioral research studies supported by NIH; *e*) develops opportunities for and supports recruitment, retention, reentry, and advancement of women in biomedical careers; and *f* ) supports research on women’s health issues. ORWH works in partnership with the NIH institutes and centers to ensure that women’s health research is part of the scientific framework at NIH and throughout the scientific community.

The ORWH announces the publication of an investigator-initiated exploratory developmental program, using the R21 grant mechanism. This trans-NIH research program funding opportunity is called Advancing Novel Science in Women’s Health Research (ANSWHR). Using the R21 grant mechanism, ORWH will fund meritorious women’s health research and sex/gender research in scientific partnership with the NIH institutes and centers.

The overall purpose of ANSWHR is to stimulate and support innovative research that will advance new concepts in women’s health research and the study of sex/gender differences. ORWH and its scientific partners across the NIH are interested particularly in encouraging extramural investigators to undertake new interdisciplinary research to advance studies on how sex and gender factors affect women’s health.

During the past several years, research reports have clearly established the importance of studying issues specific to women and female–male differences in all areas of science from basic science studies of molecular genetics to studies of epidemiology, etiology, and prevention/treatment interventions. The scientific and clinical importance of analyzing data separately for females and males is becoming more and more evident.

Because ORWH is an office within the Office of the NIH Director and does not have “grant-making authority,” the partnership between ORWH and the NIH institutes and centers will be operationalized as follows:

Applications submitted to this Program Announcement Set-Aside (PAS) will be directed to the most appropriate NIH institute or center (called primary IC), based on scientific focus, by the Center for Scientific Review (CSR). Applications will then undergo standard NIH scientific review, using established Integrated Review Groups (IRG). After scientific review and institute/center council review/approval, ORWH will provide funding for those grants selected for award. All pre-award requirements and the grant award notice will be handled by the primary IC. Scientific and grants management oversight will reside in the primary NIH institute or center for the duration of funding, with ORWH remaining actively involved in this PAS .

The scientific basis for the ANSWHR program derives from three main sources: 1) The NIH Research Priorities for Women’s Health which are reviewed and published annually on the ORWH website and is available at: http://orwh.od.nih.gov/research/priorities.html; 2) The report, Agenda for Research on Women’s Health for the 21st Century, which was developed in collaboration with the NIH and the extramural scientific and public advocacy communities; and 3) The Institute of Medicine Report, “Exploring the Biological Contributions to Human Health, Does Sex Matter?” Detailed discussion of these sources follows.

### FY 2007 NIH Research Priorities for Women’s Health

Each year, the ad hoc Research Subcommittee of the Coordinating Committee on Research on Women’s Health (CCRWH), composed of representatives from the NIH institutes and centers, considers continuing gaps in knowledge, and emerging scientific opportunities for current research priorities in women’s health. The subcommittee’s recommendations are reviewed and approved by the CCRWH and the Advisory Committee on Research on Women’s Health (ACRWH), and posted on the ORWH website, and is available at: http://orwh.od.nih.gov/research/priorities.html.

The FY 2007 NIH Research Priorities for Women’s Health are described in terms of overarching themes, areas of research interest, and special emphasis areas. The priorities signify approaches and areas for which there is a need to stimulate and encourage research on women’s health, or sex/gender factors, and the advancement of women in biomedical research careers. These research priorities are not an exclusive list of research areas important to women’s health; therefore, other innovative or significant research areas also should be considered.

The following four overarching themes are important for addressing research on women’s health: life span, sex/gender determinants, health disparities/differences and diversity, and interdisciplinary research.

#### Life span

The health of girls and women is affected by developmental, physiological, and psychological age. Women’s lives are marked by a continuum from intrauterine life to the elderly years: infancy, childhood and adolescence, menarche, reproductive life, the menopausal transition, postmenopausal years, the elderly, and the frail elderly. The lives and health status of women are influenced by many factors such as work inside and outside the home; care giving, especially to children and elder care responsibilities; reproductive status; marital status; and chronic illness. Each of these factors may influence health, disease, lifestyle, treatment choices, and response to therapy. Researchers should consider these variables in designing studies related to women’s health.

#### Sex/gender determinants

Women are characterized by both sex and gender as highlighted in the Agenda for Research in Women’s Health for the 21st Century and the Institute of Medicine report, entitled Exploring the Biological Contributions to Human Health: Does Sex Matter? In this context, the term sex refers to identification as male or female according to reproductive organs and functions assigned by chromosomal complement. Sex factors that contribute to the biological differences include chromosomes, reproduction, and hormones. Gender refers to socially defined and derived expectations and roles rooted in biology and shaped by environment and experience. Gender and sex are important considerations in most areas of research, including basic biological, psychological, social, and behavioral studies. Consideration of these variables is critical to the accurate interpretation and validation of research affecting women’s health. These variables determine how health or disease processes may differ among women or between men and women.

#### Health disparities/differences and diversity

Women are disproportionately affected by some conditions and diseases in terms of incidence, diagnosis, course, and response to treatment. Some populations of women may be at higher risk for adverse disease outcomes because of factors such as: biology, genes, culture, education, effects of poverty, access to care, quality of care, and access to opportunities for inclusion as research subjects in clinical trials and studies. Thus, clinical research should include, but not be limited to, population-specific characteristics such as cultural diversity, race/ethnicity, immigrant status, rural or inner-city residency status, effects of poverty or low socioeconomic status, sexual orientation, and physical or mental disabilities.

#### Interdisciplinary research

With increasing understanding of the interrelatedness and complexity of disease, the nature of scientific investigation is shifting to an interdisciplinary collaborative approach. Advances in women’s health can be better achieved by promoting partnerships across disciplines. Interdisciplinary approaches can integrate knowledge from multiple specialties and disciplines, thus enhancing the likelihood of defining underlying pathologic processes. Collaborations among researchers in academia, private industry, and federal settings can provide access to the latest scientific tools and technologies and expertise for women’s health research.

### Areas for Women’s Health Research

Basic, clinical and translational research should be considered in addressing priority areas in women’s health research. Some examples may include, but are not limited to: 1) Diseases and conditions that affect women. Investigate the pathogenesis and develop preventive and therapeutic interventions for acute and chronic diseases and disorders that affect women including, but not limited to, metabolic, inflammatory, endocrine, autoimmune, gastrointestinal, liver, urologic, ophthalmic, oral, reproductive, musculoskeletal, neurological, psychiatric, and cardiovascular diseases; 2) Methodological advances. Develop clinical trial methodology, including novel recruitment strategies, standardized outcome strategies, and statistical analyses that address ethical and study design issues specific to studies of women. Develop new methodologies for animal model studies of the normal development of women, and their health and diseases, including female animal models. Methodological studies related to the conceptualization, distinction, and detection of sex and gender differences in basic and clinical biomedical research; 3) Education and career development of women in science. Identify and explore factors that affect the selection and advancement of women’s careers in biomedical sciences. Implement novel education programs directed at girls and women. Promote unique programs for addressing impediments to the advancement and effective mentoring of women to senior positions in science; 4) Quality of life. Elucidate the unique sex and gender factors affecting women’s quality of life. Develop approaches to management of disease and promotion of wellness that are directed at women and their unique issues; 5) Research collaborations and partnerships. Foster special trans-NIH, trans-DHHS, and public–private research partnerships and collaborations in appropriate areas of research and career development related to women’s health.

The NIH is interested especially in fostering research in women’s health in the high-priority areas of prevention and treatment, and the biological and behavioral basis of sex and gender differences.

#### Prevention, treatment, and treatment outcomes

Increased investigation into methods to prevent conditions and diseases, or to optimize treatment, can result in significant improvements in the quality and length of women’s lives. Prevention research spans the continuum from the most basic biological studies to understanding the bases and effects of risk behaviors across the lifespan and the interventions to change them, including a focus on wellness and healthy behaviors. Examples of needed prevention and treatment research studies in women’s health include, but are not limited to: 1) Research to identify and validate biomarkers, including genetic polymorphisms, of disease risk, pathogenesis, progression, and their applications to disease prevention, early detection and treatment, including the development of novel tools; 2) Studies of the impact on health of diet, nutrition, hormones, exercise, weight patterns, toxin exposures, obesity, sex practices, mental health disorders, including eating disorders, tobacco, alcohol and drug use or abuse, occupation, violence, or trauma. Studies of the factors that are involved in disease initiation and progression, both biologic and behavioral, in order to develop effective preventive and treatment strategies; 3) Development, testing, and validation of preventive, early detection, and treatment strategies for conditions and diseases including, but not limited to, sexually transmitted diseases, cancer, coronary artery disease, stroke, obesity, diabetes, musculoskeletal disorders, pain syndromes, addictions, mental health, and chronic multisystemic diseases; 4) Studies of the effect of biological, behavioral, cultural, social, economic, and environmental factors on susceptibility to, or protection from, disease and response to treatment, such as individualized medicine and subset analyses, when appropriate.

#### Biological and behavioral basis of sex and gender differences

Whereas there has been much research to identify the function of cellular pathways and genes, research on the effects of sex as a modifier of cellular and gene function is underinvestigated. Systemic and cellular modeling of the influence of sex differences in biological pathways and systems is needed, including, but not limited to: 1) determination of sex differences that may modify the role of known cellular pathways; 2) effect of sex differences on the expression and function of genes, genetic polymorphisms, and gene defects in the risk factors, etiology, severity, and response to treatment of common diseases and those that disproportionately affect women; 3) sex and gender differences in prevention, pathogenesis, course, response to treatment, and prevention, using basic, translational, behavioral, and clinical research approaches; 4) mechanism of sex effects on gene expression and cellular and signaling pathways in healthy women, including the impact of puberty, the menstrual cycle, pregnancy, and menopause; 5) genetic, molecular, and cellular basis of action of pharmacologic agents in women, including differential effects between males and females; 6) development of novel methods of analysis to assist in discerning impact of sex in mechanisms of disease initiation, course, and response to treatment or other interventions; 7) effect of biologic and behavioral sex and gender difference on quality of life and quality of care.

### An Agenda for Research on Women’s Health for the 21st Century

The ORWH has published “An Agenda for Research on Women’s Health for the 21st Century,” A report of the task force on the NIH Women’s Health Research Agenda for the 21st Century, based on a series of four scientific workshops. This outline represents the recommendations from these national meetings examining research needs for women’s health: 1) scientific areas, 2) sex and gender perspectives throughout the life cycle; and 3) differences among populations of women throughout the life cycle. The executive summary of these outcomes is available at the following URL: http://orwh.od.nih.gov/research/resagenda.html.

### The Institute of Medicine Report, “Exploring the Biological Contributions to Human Health, Does Sex Matter?”

This report, published in 2001, includes three overarching conclusions: 1) Sex matters. Sex—that is, being female or male—is an important basic human variable that should be considered when designing and analyzing studies in all areas and at all levels of biomedical and health-related research. 2) The study of sex differences is evolving into a mature science. There is now sufficient knowledge of the biological basis of sex differences to validate the scientific study of sex differences, and to allow the generation of hypotheses. 3) Barriers to the advancement of knowledge about sex differences in health and illness exist and must be eliminated. The report states that the question of sex needs to be routinely asked in research studies, and the results, positive or negative, need to be routinely reported in order to advance our understanding of the pathogenesis of disease.

This report also discusses the terms “sex” and “gender,” and how they are sometimes used interchangeably in research literature. The report defines sex as “the classification of living things, generally as male or female according to their reproductive organs and functions assigned by their chromosomal complement, and gender as a person’s self-representation as male or female, or how that person is responded to by social institutions on the basis of the individual’s gender presentation. Gender is shaped by environment and experience.” Thus, sex is a biological construct whereas gender is a psychosocial construct. This distinction notwithstanding, epigenetic research reveals that biological factors often unfold in ways that are influenced by the environment and thus can obviate this distinction between sex and gender.

Therefore, because it is often not known *a priori* whether a female—male difference is sex-based, gender-based or both, in this program announcement “sex/gender difference” will be used generically to refer to female—male differences regardless the origins of the differences, although a search for those origins is a major focus of this announcement.

In nonhuman animal research, however, the term “sex difference” is the preferred term. Additionally, researchers are encouraged to recognize that a sex/gender difference is sometimes only a proxy for an unidentified independent variable(s). Thus, finding a sex/gender difference typically should be regarded as a first step in a search for such variables as their identification will shed light on the phenomenon under study. For review of the entire report, please see http://www.nap.edu and http://www.iom.edu/CMS/3740/5437.aspx. (http://lab.nap.edu/openbook/0309072816/html/1.html).

In addition to the three main resources presented above, other examples of areas of interest are offered as a guide to interested investigators.

Sex and gender differences include: 1) sex/gender differences in the basic behavioral, biological, and genetic mechanisms underlying diseases and conditions that predominantly affect women; and laboratory, epidemiological, and clinical studies of sex/gender differences in the etiology, onset, and progression in these disorders and conditions; 2) the genetic basis of sex/gender differences, using a variety of methodologies, such as statistics or epidemiology to ascertain heritability; identification of genetic variants through linkage, linkage disequilibrium, or association studies; identification of candidate genes using knockout or overexpression technologies in model organisms in model organisms; gene expression studies; 3) sex differences in hormonal, psychosocial, and cognitive function; 4) female—male differences in how the transition to puberty and adolescent physique characteristics influence the onset and progression of disorders and conditions that affect predominantly women; 5) sex/gender differences in the perception of pain and in efficacy of both pharmacological and nonpharmacological pain treatments; 6) design, develop, and validate preventive, early detection, and treatment strategies that use sex/gender-based theories (e.g., psychosocial, biological, developmental) and empirical findings on the risk and protective factors related to onset, progression, and treatment of diseases and conditions such as obesity, diabetes, musculoskeletal disorders, chronic pain syndromes, addictions, metabolic, inflammatory, endocrine, autoimmune, gastrointestinal, urologic, ophthalmic, oral, reproductive, neurological, psychiatric, and chronic multisystemic diseases; 7) examine the impact of key variables such as diet, nutrition, weight patterns on treatment outcomes in females and males; 8) study the effect of biological, behavioral, cultural, social, economic, and environmental factors on susceptibility to, or protection from, disease and response to treatment, such as individualized medicine and subset analyses, when appropriate.

Cross-cutting issues include: 1) address and compare stages of the life cycle, from intrauterine, to infancy, childhood, adolescence, adulthood, late adulthood, and elderly; 2) examine the role of race, ethnicity, culture, and socioeconomic class in conditions and disorders that affect women; 3) examine health disparity issues in chronic and infectious diseases and disorders that affect women; 4) study issues of females in underserved populations, e.g., rural or inner-city residence, homeless, victims of violence, migrant workers; 5) use and develop methodological approaches, including longitudinal studies, secondary data analyses, descriptive studies, novel measurement tools, and interdisciplinary approaches; 6) examine sex/gender-specific recruitment and retention issues; 7) identify triggers of inflammatory processes, roles of hormone receptor activation in inflammatory responses, and interactions of sex hormones with pathogen responses in altering inflammatory and immune responses after injury. In FY 2006, ORWH held a scientific workshop entitled, “Regulation of Inflammatory Responses: Influence of Sex and Gender.” A detailed summary from this workshop can be found at http://orwh.od.nih.gov/Inflammation_Summary.html.

### Institute/Center-Specific Research Interests and Requests

In addition to the research guidelines stated above, the following NIH institutes and centers state the following: 1) NIAID is interested in projects involving HIV and opportunistic infections; 2) NIDCR has a policy that R21 applications cannot be used for small clinical trials; 3) NIGMS is interested in research into sex differences related to the clinical areas that it supports in pharmacology, anesthesiology, and the physiological sciences related to traumatic injury and burn, and wound healing; 4) NINDS will support new research projects that assess the feasibility of a novel avenue of investigation, involve high-risk experiments that could lead to a breakthrough in a particular field, or demonstrate the feasibility of new technologies that could have major impact in a specific area. Proposals submitted under this mechanism should be limited to those with the potential for truly ground-breaking impact. NINDS will also support projects in translational research intended to discover potential targets for therapeutic intervention, to identify candidate therapeutics, or to develop assays, animal models, devices, or technologies for screening or developing therapeutics. NINDS, however, will not support R21 applications that include clinical trials or other clinical studies of potential therapies. For further details regarding NINDS use of the R21 mechanism see the NINDS website: http://www.ninds.nih.gov/funding/r21guidelines.htm. Applicants are encouraged to contact appropriate program staff within NINDS for advice about potential R21 proposals; 5) NLM is interested in topics related to biomedical informatics and knowledge management; 6) OBSSR is interested in promoting research on the behavioral and social aspects of health and illness.

Under this PAS, investigators may request funds to perform secondary data analyses of either their own data sets or other data sets that are publicly available.

This FOA will use the NIH Exploratory/Developmental Research Grant (R21) award mechanism. As an applicant, you will be solely responsible for planning, directing, and executing the proposed project.

This FOA uses “Just-in-Time” information concepts. It also uses the modular as well as the non-modular budget formats (see the “Modular Applications and Awards” section of the NIH Grants Policy Statement. Specifically, if you are submitting an application with direct costs in each year of $250,000 or less (excluding consortium Facilities and Administrative [F&A] costs), use the PHS398 Modular Budget component provided in the SF424 (R&R) Application Package and SF424 (R&R) Application Guide (see specifically Section 5.4, “Modular Budget Component,” of the Application Guide).

All foreign applicants must complete and submit budget requests using the Research & Related Budget component found in the application package for this FOA. See NOT-OD-06-096, 23 August 2006.

Exploratory/developmental grant support is for new projects only; competing renewal (formerly “competing continuation”) applications will not be accepted. Up to two resubmissions (formerly ”revisions/amendments”) of a previously reviewed exploratory/developmental grant application may be submitted. See NOT-OD-03-041, 7 May 2003.

Applicants must download the SF424 (R&R) application forms and SF424 (R&R) Application Guide for this FOA through Grants.gov/Apply.

Note: Only the forms package directly attached to a specific FOA can be used. You will not be able to use any other SF424 (R&R) forms (e.g., sample forms, forms from another FOA), although some of the “Attachment” files may be useable for more than one FOA.

For further assistance, contact GrantsInfo: 301-435-0714, (telecommunications for the hearing impaired: TTY 301-451-0088) or by e-mail: GrantsInfo@nih.gov.

The application submission dates for this PAS are available at http://grants.nih.gov/grants/funding/submissionschedule.htm. The complete version of this PAS is available at http://grants.nih.gov/grants/guide/pa-files/PAS-07-381.html.

Contacts: The complete list of agency contacts is available at http://grants.nih.gov/grants/guide/pa-files/PAS-07-381.htm.

Reference: PAS-07-381.

## NIAID International Research in Infectious Diseases (IRID) Program (R01)

The National Institute of Allergy and Infectious Diseases (NIAID) encourages the submission of R01 applications from institutions in eligible foreign countries to conduct studies and establish collaborative infectious diseases research among investigators and institutions at international sites where NIAID has significant investment in research and/or infrastructure (see below for list of NIAID programs). These grants will serve to build independent research capacity by providing direct funding to investigators who do not currently have NIAID-funded grant awards for research projects. The intent of these activities is to advance the development of local scientific expertise, build local research infrastructure, and increase collaborative research partnerships at NIAID international sites.

Collaborative projects involving investigators and institutions from international sites and the United States are particularly encouraged; a U.S. partner is not required. NIAID has a long-standing interest in and commitment to global health and international research. Topics of interest for this program are limited to research on infectious diseases, including emerging infections that are of the greatest public health significance within the applicant country (e.g., tuberculosis, malaria, HIV/AIDS, sexually transmitted diseases, diarrheal, respiratory, and enteric diseases, viral hemorrhagic fevers, viral encephalitides, parasitic diseases, and vector-borne diseases). Development of immunological, microbiological, biostatistical, epidemiological, and clinical research capacity is encouraged. Studies may be proposed on any aspect of infectious diseases (except clinical trials), including but not limited to the epidemiology, pathogenesis, immunopathogenesis of infectious diseases; epidemiologic studies to define the incidence, clinical presentations, and outcomes of diseases, identification of resistance patterns, characterization of susceptible cohorts for a particular pathogen; pilot and feasibility studies in preparation for larger studies.

NIAID supports a number of international research programs on infectious diseases, including HIV/AIDS. These include the International Centers of Excellence in Research (ICERs), International Collaborations for Infectious Disease Research (ICIDRs), the Tropical Medicine Research Centers (TMRCs), the Tuberculosis Research Program, the HIV Vaccine Trials Network (HVTN), and the HIV Prevention Trials Network (HPTN).

This International Research in Infectious Diseases (IRID) Program (R01) is intended to extend these programs by expanding the breadth of research supported at international sites and by providing support to new researchers. Applications from institutions in eligible countries where NIAID has significant investments through extramural research grants, cooperative agreements or contracts or through programs of the NIAID Division of Intramural Research are particularly encouraged. However, current NIH or NIAID funding is not a requirement.

Clinical trials will not be supported. When noninterventional clinical studies are a component of the research proposed, NIAID policy requires that studies be monitored commensurate with the degree of potential risk to study subjects and the complexity of the study. AN UPDATED NIAID policy was published in the NIH Guide on 8 July 2002 and is available at: http://grants.nih.gov/grants/guide/notice-files/NOT-AI-02-032.html. The full policy, including terms and conditions of award, is available at http://www.niaid.nih.gov/ncn/pdf/clinterm.pdf).

This Funding Opportunity Announcement (FOA) will use the NIH Research Project Grant (R01) award mechanism.

The applicant will be solely responsible for planning, directing, and executing the proposed project.

This FOA uses “Just-in-Time” information concepts. It also uses the nonmodular budget formats (see http://grants.nih.gov/grants/funding/modular/modular.htm). All foreign applicants must complete and submit nonmodular budget requests using the Research & Related Budget component found in the application package for this FOA. See NOT-OD-06-096, 23 August 2006.

At this time, it is not known if competing renewal (formerly “competing continuation”) applications will be accepted and/or if this FOA will be reissued.

Applicants must download the SF424 (R&R) application forms and the SF424 (R&R) Application Guide for this FOA through Grants.gov/Apply.

Note: Only the forms package directly attached to a specific FOA can be used. You will not be able to use any other SF424 (R&R) forms (e.g., sample forms, forms from another FOA), although some of the “Attachment” files may be useable for more than one FOA.

For further assistance, contact GrantsInfo: 301-435-0714 (telecommunications for the hearing impaired: TTY 301-451-0088) or by e-mail: GrantsInfo@nih.gov.

The application receipt dates for this PAR are 26 September 2007 and 2008. The complete version of this PAR is available at http://grants.nih.gov/grants/guide/pa-files/PAR-07-376.html.

Contacts: The complete list of agency contacts is available at http://grants.nih.gov/grants/guide/pa-files/PAR-07-376.html.

Reference: PAR-07-376.

